# HierCC: a multi-level clustering scheme for population assignments based on core genome MLST

**DOI:** 10.1093/bioinformatics/btab234

**Published:** 2021-04-06

**Authors:** Zhemin Zhou, Jane Charlesworth, Mark Achtman

**Affiliations:** Warwick Medical School, University of Warwick, Coventry CV4 7AL, UK; Warwick Medical School, University of Warwick, Coventry CV4 7AL, UK; Warwick Medical School, University of Warwick, Coventry CV4 7AL, UK

## Abstract

**Motivation:**

Routine infectious disease surveillance is increasingly based on large-scale whole-genome sequencing databases. Real-time surveillance would benefit from immediate assignments of each genome assembly to hierarchical population structures. Here we present pHierCC, a pipeline that defines a scalable clustering scheme, HierCC, based on core genome multi-locus typing that allows incremental, static, multi-level cluster assignments of genomes. We also present HCCeval, which identifies optimal thresholds for assigning genomes to cohesive HierCC clusters. HierCC was implemented in EnteroBase in 2018 and has since genotyped >530 000 genomes from *Salmonella*, *Escherichia/Shigella*, *Streptococcus*, *Clostridioides*, *Vibrio* and *Yersinia*.

**Availability and implementation:**

https://enterobase.warwick.ac.uk/ and Source code and instructions: https://github.com/zheminzhou/pHierCC

**Supplementary information:**

Supplementary data are available at *Bioinformatics* online.

## 1. Introduction

Following its introduction in 2011 (Mellmann *et al.*, 2011), core genome multi-locus sequence typing (cgMLST) was widely adopted as a scalable, portable and easily communicable genotyping solution for the genome-based, routine surveillance of bacterial pathogens (Jolley *et al.*, 2012; Jones *et al.*, 2019; Moura *et al.*, 2016). In a cgMLST scheme, bacterial genomes are assigned to sequence types (STs) consisting of 1000s of integers, which each represents a distinct sequence variant (allele) of a soft core gene (Zhou *et al.*, 2020). A pairwise comparison of allelic differences between STs approximates the genetic distance between genomes and can be used for downstream phylogenetic analyses (Zhou *et al.*, 2018). However, STs are arbitrary constructs, and natural bacterial populations can each encompass multiple, related STs. 

Several single-level clustering schemes have been applied to cgMLST schemes to extract single-level clusters (SCs) from hierarchical clustering. Such SCs were equated with sub-lineages in *Listeria* (Moura *et al.*, 2016) or single source outbreaks of *Salmonella* serovar Enteritidis (Coipan *et al.*, 2020). However, because SC schemes identify only one clustering level, they ignore the wide spectrum of genetic diversities and the existence of multiple hierarchical clusters (HCs) of natural populations.

Unlike MLST schemes, multiple multi-level clustering schemes for bacterial pathogens exist that are based on core genomic single nucleotide polymorphisms (SNPs). For example, SnapperDB assigns *Salmonella* genomes to so-called SNP addresses, consisting of seven hierarchical single-linkage clusters based on SNP distances (Dallman *et al.*, 2018). Similarly, genomes of *Yersinia pestis* or *Salmonella* Typhi are assigned to one of multiple levels of sub-lineages based on their placement in a phylogeny (Morelli *et al.*, 2010; Wong *et al.*, 2016). However, SNP-based approaches are restricted to relatively uniform clades because, unlike cgMLST schemes that can extend to the genus level (Zhou *et al.*, 2020), the SNP calls and phylogenetic reconstructions become less reliable at higher levels of intra-genus diversity.

Here we present pHierCC, a pipeline that defines a scalable HierCC scheme that assigns bacterial genomes in real-time to multi-level clusters spanning a wide spectrum of genetic diversities. We also present HCCeval, which identifies optimal levels from the HierCC scheme, and yields multi-level HCs that likely represent hierarchical natural bacterial populations up to the species level.

## 2. pHierCC workflow

**Fig. 1.**
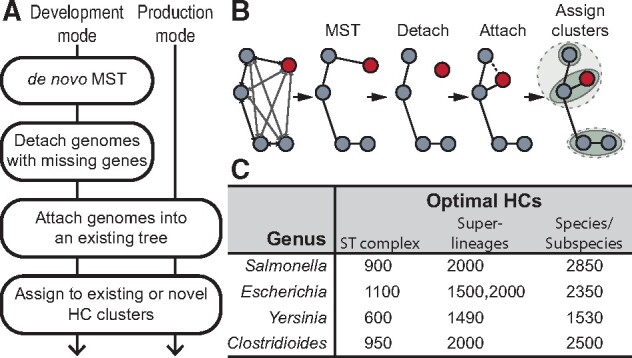
(**A**) The workflows of pHierCC in development or production mode. (**B**) Cartoon of the workflow in development mode. The node in red indicates a genome that carries numerous missing genes. (**C**) The optimal HC levels identified by HCCeval in four EnteroBase databases

pHierCC firstly calculates a minimum spanning tree (Fig. 1) based on a distance metric that minimizes the topological distortion due to missing genes (Supplementary Text). The resulting tree is used to assign every genome to clusters in multiple HCs. Subsequent assignments by pHierCC are performed in ‘production mode’. In production mode, new genomes that are equidistant to multiple clusters are automatically assigned to the oldest cluster (lowest cluster designation) in order to ensure the long-term stability of nomenclature designations (Supplementary Text).

## 3. Evaluation of optimal HCs with HCCeval

We developed an additional tool, HCCeval, to identify the optimal HCs that distinguish between natural, genetically discrete populations within a given set of STs and their corresponding HierCC assignments. HCCeval compares both normalized mutual information (NMI) (Kvalseth, 2017) and Silhouette scores (Rousseeuw, 1987) at each HC level with clustering at other levels as a measure of their relative stability (Supplementary Fig. S1). NMI measures the similarity of clustering by a given pair of hierCC levels as a harmonic mean of homogeneity and completeness between the two. HCCeval provides a heat map of these NMI scores in which sets of continuous HC levels that identify highly similar clusters (NMI ≥0.9) form visually recognizable blocks of stable HC clusters that do not change dramatically at slightly different thresholds of allelic differences. Silhouette evaluates cluster cohesiveness by comparing the internal pairwise similarities of each genome in a cluster with its similarities to genomes from other clusters. The greatest silhouette score for each stable NMI block is likely to correspond to an HC level that is optimal for identifying natural populations.

## 4. Implementation in EnteroBase

In 2018, EnteroBase calculated an initial set of HierCC HCs in development mode for representative genomes from *Salmonella, Escherichia, Yersinia* and *Clostridioides.* These representatives consisted of one genome per ribosomal MLST type, and their HCs were evaluated by HCCeval. Visual inspection of the results identified three to four stable blocks of HCs for each genus (Supplementary Fig. S1 and Frentrup *et al.*, 2019). The highest HC blocks (HC1530–HC2850 depending on genus) distinguish subspecies or species (Fig. 1C) (http://enterobase.readthedocs.io/en/latest/HierCC_lookup.html). The lowest blocks (HC600–HC1100) identify ST complexes or comparable populations defined by 7-gene MLST schemes (Frentrup *et al.*, 2019; Zhou *et al.*, 2020). Additionally, EnteroBase publishes HC assignments for HC0, HC2, HC5, HC10, HC20, HC50, HC100, HC200 and HC400 even though these do not form blocks in the NMI comparisons. In 2020, HierCC schemes for *Vibrio* and *Streptococcus* were added, but their final evaluations are still in progress.

Experience has indicated that some infectious outbreaks (European Centre for Disease Prevention and Control and European Food and Safety Authority, 2020; Jones *et al.*, 2019) or recent local transmissions (Frentrup *et al.*, 2019; Zhou *et al.*, 2020) are each associated with a cluster defined by one of these low diversity HC levels. However, our current experience indicates that clusters of genetically closely associated genomes may not necessarily represent traditional transmission chains because several have now been identified that have spread globally and continued to exist over decades. We therefore recommend that the final definition of a transmission chain continue to be based on epidemiological criteria in addition to genetic similarities. We also recommend that genetic analyses should be performed over a range of HCs in order to ensure that populations are truly distinct.

## 5. Conclusions

In this article, we introduce pHierCC, a pipeline that defines HierCC, a scalable, fine-grained, incremental clustering scheme for bacterial genomes based on their cgMLST allelic profiles. HierCC was integrated into EnteroBase in 2018, and pHierCC has currently assigned >530 000 genomes from *Salmonella*, *Escherichia/Shigella*, *Streptococcus*, *Clostridioides*, *Vibrio* and *Yersinia* into 12–13 multi-level clusters ranging from sub-clonal variation to species. pHierCC is also available as a stand-alone package that can be used for any bacterial genus with a cgMLST scheme and where large numbers of genomes are available for the initial assignments.

## Supplementary Material

btab234_Supplementary_Data
