# Supplementary material for: HierCC: a multi-level clustering scheme for population assignments based on core genome MLST
Source: Bioinformatics. 2021 Apr 6;37(20):3645–6. doi: 10.1093/bioinformatics/btab234 (PMC8545296; doi:10.1093/bioinformatics/btab234)

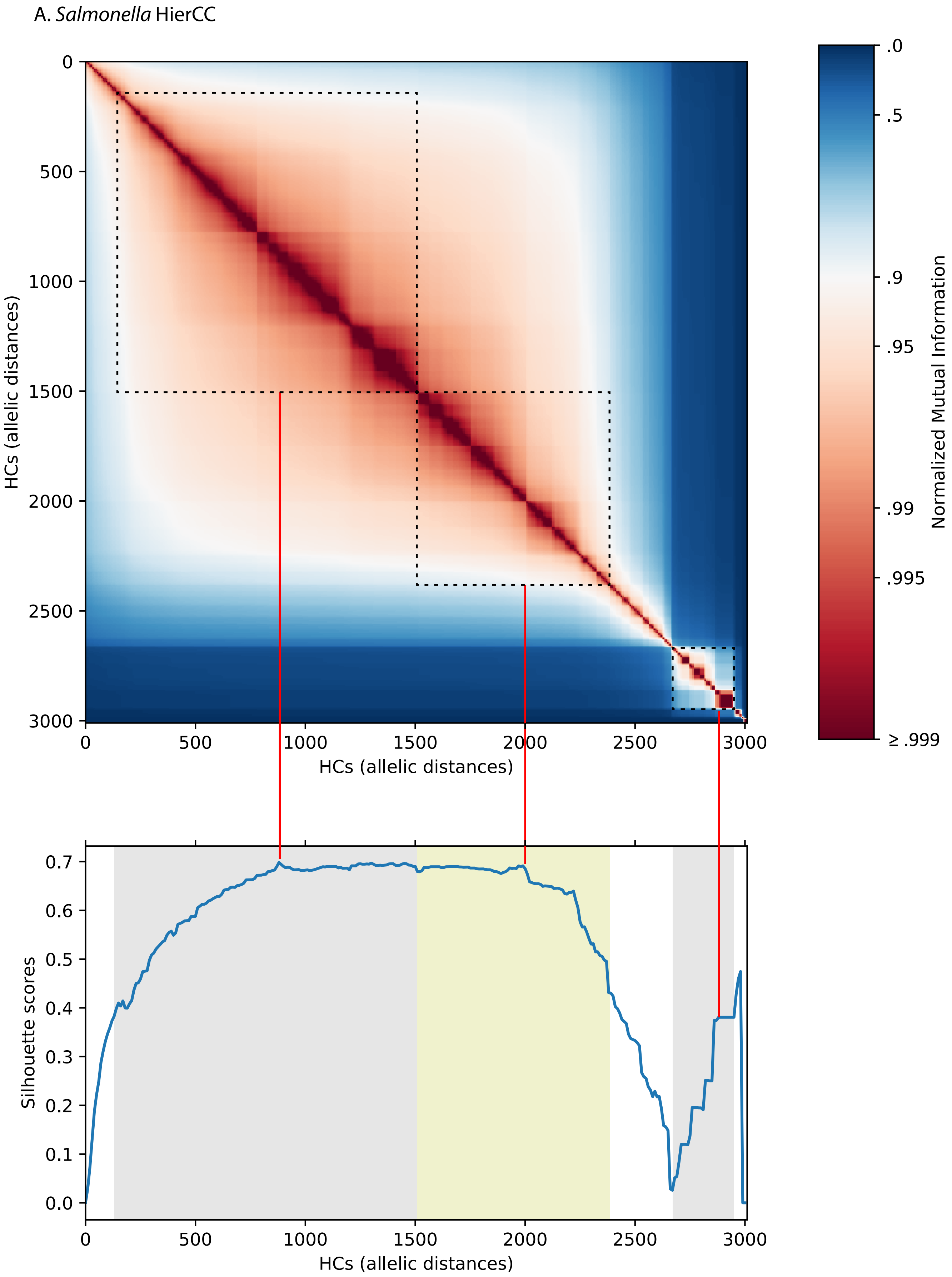

Supplementary Figure 1. Statistical evaluation of HierCC at all levels for EnteroBase databases of *Salmonella* (A), *Escherichia/Shigella* (B) and *Yersinia* (C). The heat plot at the top of every figure shows pairwise similarities of clusterings from different HC levels as Normalized Mutual Information (NMI) scores, which each measures harmonic mean of the homogeneity and completeness between two clusterings. The line plot at the bottom shows the cohesiveness of the clustering at every HC level as a measure of silhouette score, which compares the similarity of a genome to its own cluster with its similarities to other clusters. The dotted boxes and the shaded area indicates blocks of stable HC levels that result to similar (stable) clusterings, and the red lines indicate chosen HC levels of maximum cluster stability for each HierCC scheme.

B. *Escherichia/Shigella* HierCC

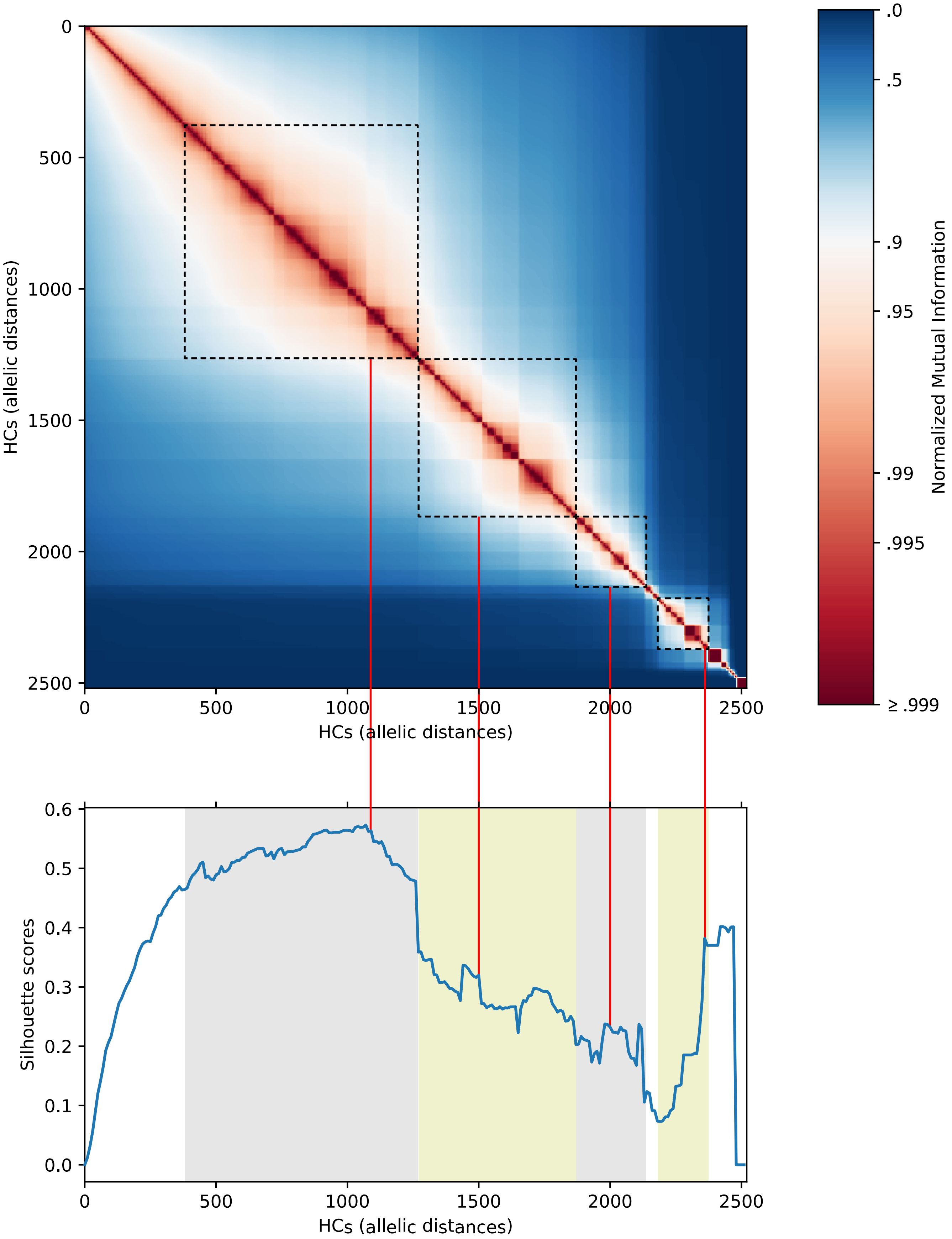

C. *Yersinia* HierCC

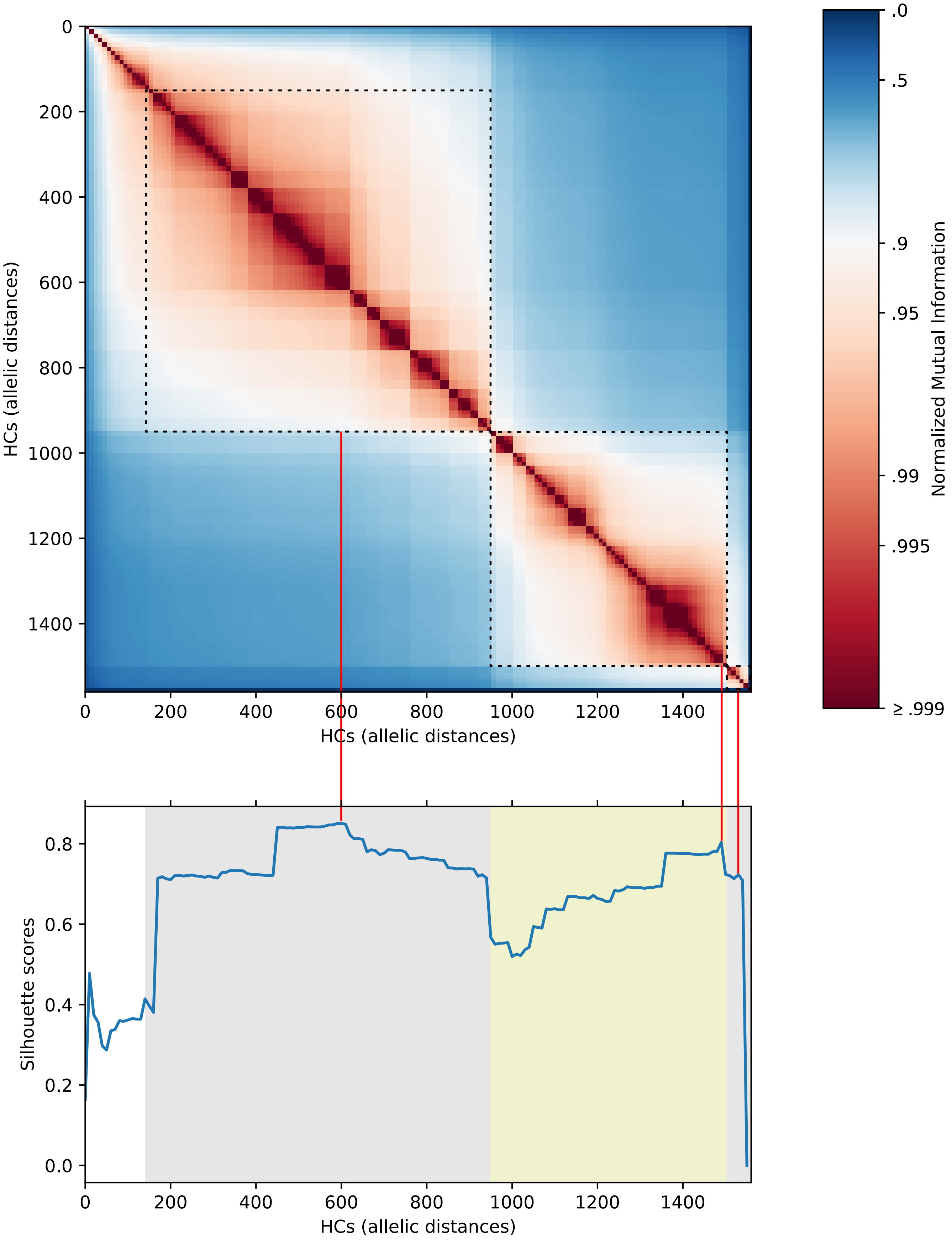

Supplement: btab234_Supplementary_Data [file btab234_supplementary_data.zip › FigS1 v3.pdf]
