# Supplementary material for: HierCC: a multi-level clustering scheme for population assignments based on core genome MLST
Source: Bioinformatics. 2021 Apr 6;37(20):3645–6. doi: 10.1093/bioinformatics/btab234 (PMC8545296; doi:10.1093/bioinformatics/btab234)
Supplement: btab234_Supplementary_Data [file btab234_supplementary_data.zip › SupplementaryText V2.2.docx]

| Supplementary Text: the workflow of pHierCC  Zhemin Zhou^*^, Jane Charlesworth and Mark Achtman  Warwick Medical School, University of Warwick, Gibbet Hill Road, Coventry, CV4 7AL, United Kingdom  *To whom correspondence should be addressed.  **Contact:** zhemin.zhou@warwick.ac.uk. **Current address:** Pasteurien College, Soochow University, Suzhou, China |
| --- |

## *de novo* MST construction (Development mode only)

pHierCC uses the Python SciPy package to generate a minimum spanning tree (MST) from a distance matrix of the numbers of differing alleles in pairwise comparisons (Fig. 1). However, excessive missing genes can artificially distort a distance matrix toward conserved, smaller genes because larger genes provide more opportunities for deletion. We arbitrarily chose to deal separately with that tiny subset of all pairs of genomes (<1%) which differ by more than 3% of missing data in order to minimize these distorting effects. (The default value for pHierCC can be stipulated with the runtime parameter “--allowed_missing”). Given a cgMLST scheme with *n* core genes, such problematical pairs were identified by calculating s, a minimum number of shared genes between pairs of genomes as:

$$s=m-0.03*n$$

where $m$ is the number of core genes in the more complete genome. The allelic distance *d* between two genomes is calculated as:

$d=\left\{ \begin{aligned} \left\lfloor n*{d_{0}}/{s_{0}}+0.5 \right\rfloor, s_{0}\geq s \\ \left\lfloor n*{(d_{0}+s-s_{0})}/s+0.5 \right\rfloor, s_{0}<s \end{aligned} \right.$ (1)

where *s_0_* is the number of core genes common to both genomes and *d_0_* is the number of different alleles. In the second case, pHierCC elevates the allelic distances between the two genomes, thereby increasing the likelihood that genomes with more missing data remain outliers and do not join tight clusters.

After completion of the MST, pHierCC actively removes genomes with >3% of missing genes from the MST, and deals with them in the next step.

## Attach nodes to MST

Given a detached genome *q* and a genome *r* in the MST, pHierCC calculates a directional distance from *r* to *q* as:

$d_{r\to q}^{'}=\left\{ \begin{aligned} \left\lfloor n*{d_{0}}/{s_{0}}+0.5 \right\rfloor, s_{0}\geq s' \\ \left\lfloor n*{(d_{0}+s'-s_{0})}/{s'}+0.5 \right\rfloor, s_{0}<s' \end{aligned} \right.$ (2)

Equation 2 only differs from equation 1 by its use of *s’*, which is calculated as:

$$s’=m_{q}-0.03*n$$

where *m_q_* is the number of core genes in *q*. Equation 2 may give a lower distance measure than equation 1 when there are more missing genes in *q* than in *r*. An edge is then drawn between *q* and a genome *r* in the tree that minimizes $d_{r\to q}^{'}$, unless multiple similar STs are equidistant, in which case the edge is to the genome with the smallest (oldest) ST designation .

## Assign each genome to hierarchical clusters

pHierCC applies every threshold $c\in\{0..n\}$ to a copy of the MST by removing all edges with greater distances than that threshold. All remaining, discrete subtrees constitute the clusters for that threshold, and are designated by the smallest ST designations within each cluster. As a result, each genome is assigned to *n +1* clusters, from Hierarchical Cutoff 0 (HC0) which groups together genomes that have no allelic difference except for missing data, to HC*n* which consists a single cluster of all genomes.

## Production mode

When the runtime parameters trigger the pHierCC pipeline to load an optional existing tree together with new genomes, pHierCC changes to “production mode”, and only performs the workflows in sections 2 and 3 above, without recalculation of an MST (Fig. 1A). In production mode, cluster assignments of previously assigned genomes are not modified by subsequent attachment of new genomes to the tree. Instead, ambiguous joins of every new genome multiple equidistant clusters are automatically resolved by assigning that new genome into the older cluster with the smaller cluster number. One consequence of this behavior is that it precludes the gradual merging of clusters which is so typical of ST complexes in SC assignments of 7-gene MLSTs. However, we note that the resulting HierCC tree is no longer an MST, and genetically closely related genomes may be assigned to distinct clusters.
